# Combined radiation and *Listeria* immunotherapy induces cytotoxic immunity that correlates with improved outcome in dogs with osteosarcoma

**DOI:** 10.1016/j.omton.2026.201243

**Published:** 2026-05-21

**Authors:** Nicola J. Mason, Josephine Gnanandarajah, Martha MaloneyHuss, Jennifer Reetz, Kimberly A. Agnello, Falon Gray, Julie Engiles, Lauren Olenick, Andrew Hart, Douglas H. Thamm, Yvonne Paterson

**Affiliations:** 1 Department of Pathobiology, School of Veterinary Medicine, University of Pennsylvania, Hill Pavilion, 380 South University Avenue, Philadelphia, PA 19104, USA; 2 Kashiv BioSciences, LLC. 20 New England Ave, Piscataway, NJ 08854, USA; 3 Integrity Veterinary Center, Northampton, MA 01060, USA; 4 Department of Clinical Sciences and Advanced Medicine, School of Veterinary Medicine, University of Pennsylvania, 3900 Delancey Street, Philadelphia, PA 19104, USA; 5 Bristol Myers Squibb, Cambridge, MA 02141, USA; 6 Flint Animal Cancer Center, College of Veterinary Medicine and Biomedical Sciences, Colorado State University, 300 West Drake Road, Fort Collins, CO 80523, USA; 7 Department of Microbiology, Perelman School of Medicine, University of Pennsylvania, Philadelphia, PA 19104, USA

**Keywords:** immunotherapy, radiation therapy, combination therapy, HER2, osteosarcoma, cytotoxicity, canine, pre-clinical, Listeria, comparative

## Abstract

Ionizing radiation acts synergistically with immunotherapy by inducing immunogenic cell death and modulating the tumor microenvironment, promoting anti-tumor immune responses. In this pilot study, the safety and effectiveness of palliative radiation with a *Listeria* expressing *HER2* (Lm-LLO-HER2), on delaying primary tumor progression in immunocompetent dogs with osteosarcoma was evaluated and correlative immune response biomarkers explored. Fifteen dogs with treatment naive, appendicular osteosarcoma, without evidence of metastatic disease received palliative radiation followed by Lm-LLO-HER2 immunotherapy every 3 weeks for an initial 8 doses then booster treatments. Treatment was well tolerated and delayed primary tumor progression and prolonged overall survival in 5/15 dogs. Peripheral blood mononuclear cell transcriptomic profiling revealed that baseline immune status and immune response to Lm-LLO-HER2 distinguished long-term from short-term survivors. Genes associated with Hedgehog signaling, senescence, DNA damage repair, and cell cycle were downregulated when compared to baseline in long-term survivors. These findings support further exploration of combination palliative radiation and *Listeria*-based immunotherapy in osteosarcoma and suggest that baseline immune status may determine clinical and immunological responses to combination therapy. Pet dogs with osteosarcoma represent a parallel population to pediatric osteosarcoma patients. These findings may inform future pediatric osteosarcoma clinical trials and patient stratification.

## Graphical abstract

**Figure:**
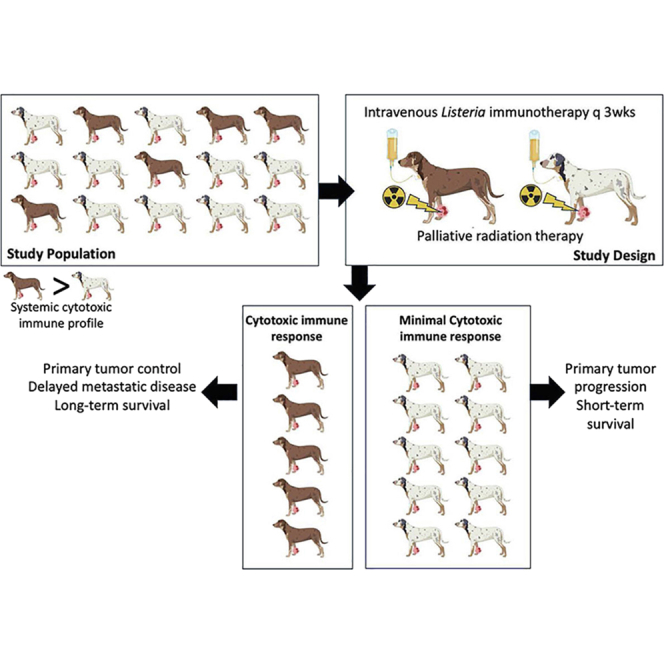

## Teaser

Local radiation combined with a HER2 expressing *Listeria* induced systemic cytotoxic immune responses that correlated with local tumor control, delayed metastatic disease onset, and prolonged survival in dogs with spontaneous, primary, appendicular osteosarcoma. Baseline and post-treatment immune activity distinguished long from short-term survivors and may aid in pediatric osteosarcoma stratification.

## Introduction

Harnessing the specificity and cytotoxic capabilities of the immune system to eliminate neoplastic cells and generate immunological memory represents a powerful strategy to induce durable remissions in cancer patients. The overarching goal of immunotherapy is to induce a broad and self-propagating anti-tumor T cell response that controls primary and metastatic disease and prevents future relapse.^1^ Therefore, combination therapies that address different aspects of the cancer-immunity cycle may offer the best chance of immunotherapeutic success, particularly in solid tumors. Ionizing radiation induces immunogenic tumor cell death which provides an *in situ* vaccine effect, due to release of tumor antigens and damage-associated molecular pattern molecules (DAMPs), such as ATP and HMGB1 that mature dendritic cells (DCs) and promote anti-tumor T cell responses. Radiation therapy (RT) has a marked immunomodulatory effect on the TME, promoting T cell infiltration through induced expression of chemokines and increased expression of ICAM-1 on vascular endothelium. Low doses of RT can reprogram tumor-associated macrophages (TAM) toward a proinflammatory M1 phenotype and activate DNA sensing pathways such as cGAS and STING in tumor cells and immune cells, leading to the release of type I IFN which promotes innate and adaptive immune responses (reviewed in Gao and Zhang^2^). Through these effects, RT expands the TCR repertoire and broadens the immune response against tumor cells, addressing the genetic heterogeneity of solid tumors responsible for tumor escape after target immunotherapies.^3^ Given the permissive effects of RT on the tumor microenvironment (TME) and its ability to promote anti-tumor immunity, it is actively being explored in combination with immunotherapies to promote effective anti-tumor T cell responses, enhance local tumor control, and prevent metastatic disease.

*Listeria monocytogenes* (*Lm*) is an intracellular bacterium that is a potent inducer of innate and adaptive immunity and an effective biological vector to deliver tumor-associated antigens (TAA) to antigen-presenting cells.^4^^,^^5^ The ability of *Lm* to secrete the pore forming lysin, listeriolysin O (LLO) enables it to escape from the phagolysosome into the cytosol of the cell where it undergoes proteasomal degradation and is presented on MHCI to prime CD8^+^ T cells. TAA can be fused to LLO and transfected into highly attenuated strains of engineered *Listeria* to generate tumor vaccines that induce potent, antigen-specific CD4^+^ and CD8^+^ T cell responses.^6^^,^^7^^,^^8^ Furthermore, the adjuvant effect of attenuated Lm-LLO vectors boosts existing T cell responses against endogenous epitopes, promoting epitope spreading in mouse models and in human clinical trials,^9^^,^^10^^,^^11^ and epitope spreading in response to immunotherapies is correlated with therapeutic response.^12^

Lm-LLO-HER2 (OST31-164, formally ADXS31-164) is a highly attenuated *Listeria monocytogenes* that is engineered to express a chimeric, recombinant human HER2 molecule fused to LLO.^13^ Lm-LLO-HER2 causes antigen-specific regression of established HER2-positive cancers in multiple mouse models and prevents the establishment of metastatic lesions in aggressive metastatic mouse models of HER2^+^ malignancies.^6^^,^^7^ In a small pilot study, Lm-LLO-HER2 administration to dogs with HER2^+^ osteosarcoma (OSA) following amputation and chemotherapy was safe, broke tolerance to HER2, and delayed both metastatic disease and prolonged overall survival time (OST).^14^ In a more recent larger canine study, the ability of Lm-LLO-HER2 to induce innate and adaptive immune responses correlated with clinical outcome.^15^ Importantly, *Listeria* vectors increase cytotoxic CD8^+^ T cell numbers, and reduce regulatory T cells (Tregs) and myeloid derived suppressor cells (MDSCs) within the TME, enabling primed tumor-specific T cells to function more effectively.^16^^,^^17^ Studies evaluating combination of *Lm* vaccines and RT in mouse models of melanoma and prostate cancer, showed that the combination led to an increase in activated T cells in the TME and an increase in antigen-specific T cells within the spleen respectively and showed functional superiority over either modality alone.^18^^,^^19^

In this study, we sought to determine the safety, efficacy, and clinical outcomes of combination palliative RT (pRT) and Lm-LLO-HER2 on primary tumor progression and development of metastatic disease in dogs with spontaneous, appendicular OSA. Canine OSA recapitulates many aspects of pediatric OSA, including high genetic instability and histologic heterogeneity, aggressive local disease and early metastases, and comparable surgical, chemotherapeutic, and radiation treatment options. Therefore, dogs with OSA are widely recognized as a clinically relevant, spontaneous, immune competent, and parallel patient population.^20^^,^^21^^,^^22^^,^^23^ The median survival time of dogs with appendicular OSA treated with amputation and chemotherapy is 10 months. Approximately 90%–95% of dogs have micrometastases at diagnosis and despite therapy; most dogs are euthanized due to progressive metastatic disease. Although ionizing radiation is not a mainstay of primary tumor treatment in the pediatric setting, it is widely employed along with analgesics for palliative care in dogs with OSA that do not undergo amputation and chemotherapy, a decision that is largely owner driven and based on cost, concurrent orthopedic issues, and/or perceived quality of life issues following amputation. In these instances, a standardized, palliative intent, RT protocol consisting of hypofractionated RT (8Gy on 2 consecutive days) with or without concurrent bisphosphonate treatment has been reported to provide pain relief and improve quality of life. However, most dogs rapidly succumb to progressive, local disease with or without metastases within 3–5 months.^24^^,^^25^ In both species the epidermal growth factor receptor HER2 is expressed within malignant osteoblasts and represents a potential immune therapeutic target for T cells.^26^^,^^27^^,^^28^^,^^29^ We hypothesized that combination pRT plus Lm-LLO-HER2 would safely delay primary tumor progression, delay or prevent metastatic disease, and prolong OST in dogs with appendicular OSA that do not undergo amputation and chemotherapy. We further hypothesized that combination therapy would promote a cytotoxic immune response that would correlate with clinical outcome.

## Results

Twenty-eight dogs were screened for trial eligibility and 16 dogs fulfilled the eligibility criteria. Twelve screened dogs were ineligible based on the presence of suspected metastatic disease (*n* = 2), concurrent systemic disease (*n* = 1), pathological fracture (*n* = 2), non-diagnostic biopsy sample or other tumor type (*n* = 3), advanced primary disease and local lymph node involvement (*n* = 2), and owner dissent (*n* = 2). One eligible dog received 2 doses of pRT but developed a panhypoproteinemia suspected secondary to non-steroidal anti-inflammatory drug (NSAID)-associated gastric ulceration. This dog was removed from the study before receiving Lm-LLO-HER2 and was therefore not considered evaluable. The age, breed, sex, primary tumor location, subtype, ALKP status, and HER2 expression score (where available) of the 15 evaluable dogs are shown in Table S1.

**Figure 1:**
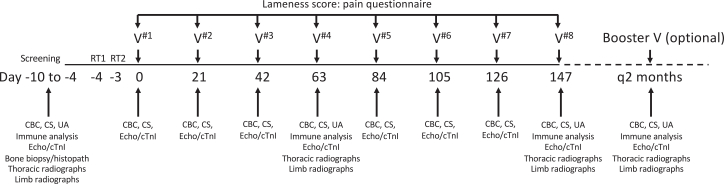
Pilot study outline and timeline Dogs were screened 4 to 10 days prior to receiving immunotherapy to confirm general health status and histopathological diagnosis of OSA. Dogs received 2 doses of 8Gy palliative radiation (RT1 and RT2) on consecutive days followed by the first of 8 scheduled Lm-LLO-HER2 treatments (V#1–8). CBC, complete blood count; CS, chemistry screen; UA, urinalysis; cTnI, cardiac troponin I.

The number of days between tumor biopsy and the first dose of radiation ranged from 0 (biopsy and radiation on the same day, *n* = 2) to 32 days, (mean, 13.53 days; median, 10 days). Fifteen dogs each received a total of 16 Gy pRT, divided into 2 doses of 8 Gy given on 2 consecutive days. Dogs received the first of eight intended doses of 3.3 × 10^9^ CFU of Lm-LLO-HER2 2 to 4 days after the second RT fraction (mean, 3.4 days; median 3 days) (Figure 1). Five dogs completed the intended 8 Lm-LLO-HER2 doses, and 4 of these 5 dogs received additional booster doses (Table S1).

### Adverse effects of combination RT plus Lm-LLO-HER2

**Figure 2:**
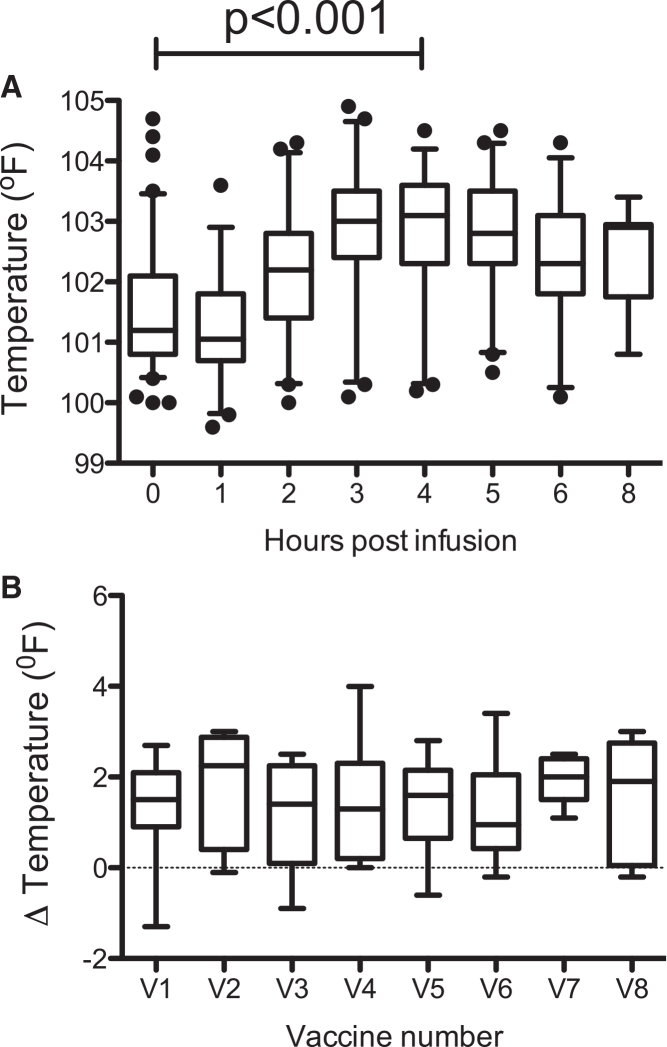
Effects of Lm-LLO-HER2 on body temperature (A) Temperatures for all dogs receiving Lm-LLO-HER2 were measured at each time point at each vaccination. (B) The change in temperature between 0 and 4 h was plotted for all dogs for each vaccination. Circles represent values lower than the 5^th^ percentile and higher than the 95^th^ percentile.

3/15 evaluable dogs experienced wound dehiscence after incisional biopsy following RT. Two of these dogs received their first dose of RT on the same day as biopsy and required medical management of wound dehiscence. The remaining dog received RT 7 days post-biopsy and required surgical management of wound dehiscence. All dogs tolerated Lm-LLO-HER2 well with only transient, mostly low-grade toxicities, including fever, nausea, and vomiting, that occurred on the day of administration as previously described (Table S2).^14^ Mild to moderate increases in body temperature occurred and peaked 4 h after each vaccine administration (Figure 2A). The timing and degree of hyperthermia remained consistent over the course of vaccines (Figure 2B), and there was no correlation between magnitude of temperature increase after the first Lm*-*LLO-HER2 administration and OST (data not shown). Hypotension, reported as a dose-limiting toxicity in humans receiving an HPV-E7 targeting recombinant *Lm*, was mild and transient in 1 dog and did not require intervention.^30^ Neither incidence nor grade of toxicities increased with additional Lm-LLO-HER2 administrations (data not shown). Complete blood count (CBC) and chemistry screen (CS) were performed at the time of each Lm-LLO-HER2 administration. No significant sustained changes were observed in CBC or CS parameters over the course of scheduled Lm-LLO-HER2 administrations (data not shown).

HER2 is expressed in both the human and canine myocardium, and immune targeting of this receptor in humans has been reported to cause cardiotoxicity.^31^^,^^32^ Cardiac status was evaluated at baseline and at every Lm-LLO-HER2 administration time point thereafter by echocardiography and cardiac troponin I concentrations. No significant, sustained changes in cardiac parameters were identified in any dog (Figure S1). One dog developed an asymptomatic, accelerated idioventricular rhythm within 4 h of her third dose of Lm-LLO-HER2. No treatment was given, and an electrocardiogram performed 4 days later showed a normal sinus rhythm. The dog developed progressive disease and did not receive a fourth vaccine. Similar low grade, transient, asymptomatic arrhythmias were identified following Lm-LLO-HER2 administration after amputation and carboplatin treatment in 2 dogs in prior study^14^; interestingly all three dogs experiencing cardiac arrhythmias following Lm-LLO-HER2 treatment were Rottweilers.

### Clinical outcome and overall survival times

**Figure 3:**
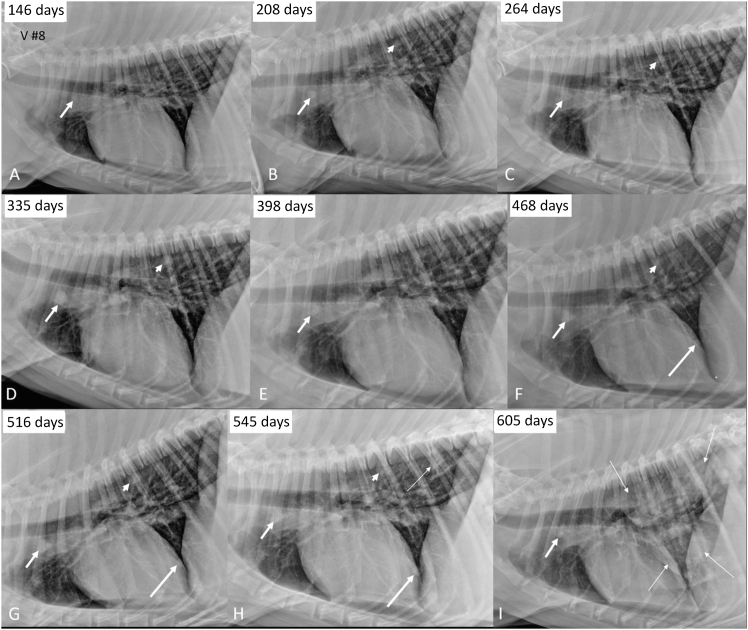
Series of thoracic radiographs of dog 005 showing evidence of suspected pulmonary metastatic disease (A) One nodule was first identified at the time of the 8^th^ Lm-LLO-HER2 treatment, day 156 (arrow). (B) Nodule enlarged within 2 months (arrow) with a new second indistinct nodule in the caudal lung (arrowhead). (C and D) Both nodules have progressed. (E) Original nodule has improved (arrow) and second nodule is not clearly identified. (F) Both nodules have progressed with a third nodule in the caudoventral lung (long arrow). (G and H) Continued nodule progression with at least one additional nodule at 555 days (long thin arrow). (I) Progressive disease with multiple ill-defined masses in the caudal lung (long thin arrows). The dog did not receive a booster vaccine at the first 2-months post treatment time point due to a lack of IACUC approval for repeat vaccines at that time. The dog received Lm-LLO-HER2 again on day 274, 345, and 408. Only right lateral radiographs are shown but left lateral and VD views confirm these findings.

**Figure 4:**
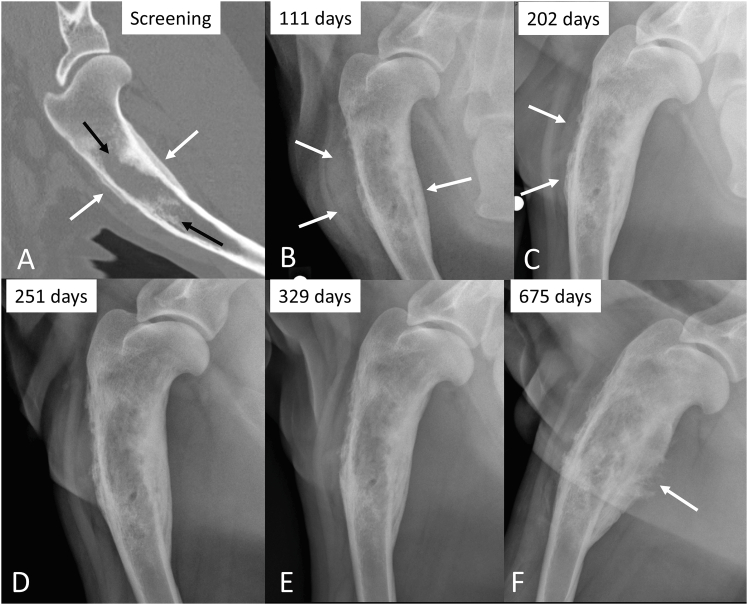
CT image at screening and follow up series of thoracic limb radiographs of dog 003 showing evidence of early progression then improvement in primary lesion and delayed progression of disease over 1 year and 10 months (A) Aggressive bone lesion in the proximal humerus with intramedullary osteolysis > osteoproliferation (black arrows) and smooth periosteal proliferation (white arrows). (B) Progressive disease with increased cranial soft tissue thickening, and increased cranial/caudal periosteal reaction, which is now mildly irregular along the cranial aspect (white arrows). (C) Improved soft tissue thickening and stable periosteal reaction (white arrows). (D and E) Stable disease. (F) Progressive disease with thicker and now spiculated periosteal reaction (white arrow).

**Figure 5:**
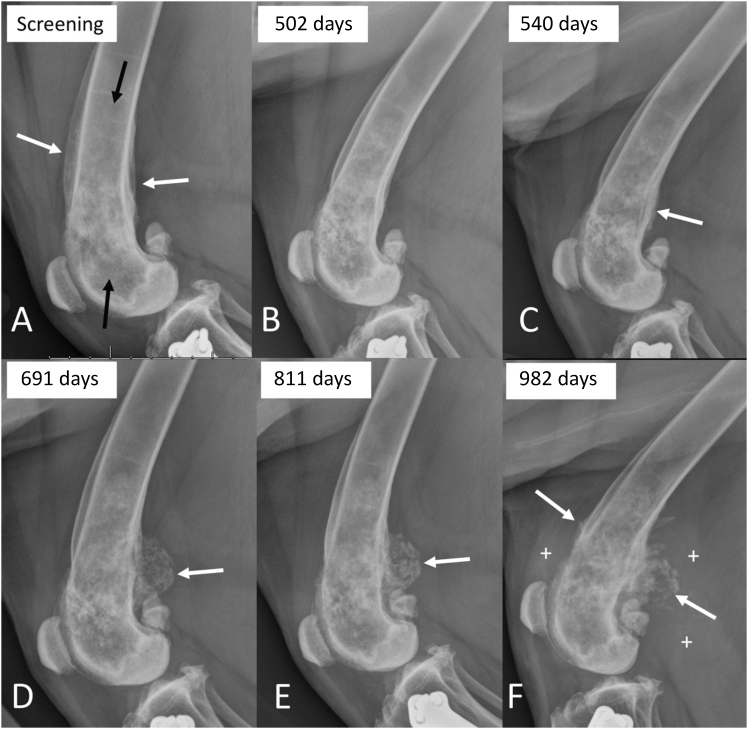
Series of pelvic limb radiographs of dog 007 showing evidence of early improvement in primary lesion and delayed progression of disease over 2 years and 8 months (A) Aggressive bone lesion in the distal femur with intramedullary osteolysis and osteoproliferation (ill-defined lesion between black arrows) and smooth periosteal reaction (white arrows). (B) Improved intramedullary and periosteal bone changes. (C) Progressive disease with early caudodistal cortical lysis, focal irregular periosteal reaction (white arrow) and impression of increased intramedullary osteolysis. (D–F) Continued progressive disease with further cortical and intramedullary lysis, progressive intramedullary osteoproliferation, and progressive amorphous to irregular periosteal reaction (white arrows) and soft tissue thickening (+).

To determine the effects of combination pRT and Lm-LLO-HER2 on the primary tumor and the ability to prevent pulmonary metastatic disease, radiographs of the affected limb and thorax were performed at the time of screening and at the fourth (day 63) and eighth (day 147) Lm-LLO-HER2 treatments and at 2-months intervals thereafter (Figure 1). Lameness scores were assigned to each dog at each scheduled visit. Two dogs were euthanized before their fourth treatment: one due to a pathologic fracture and the other due to local disease progression and pulmonary metastatic disease. Of the thirteen dogs that survived to have radiographs taken at the time of their fourth treatment, seven had radiographic evidence of local disease progression and one of these had pulmonary metastatic disease. Two dogs with local disease progression at this time underwent amputation and follow up carboplatin therapy with OST of 311 days and 154 days. Six dogs remained on study to have radiographs taken at their eighth Lm-LLO-HER2 treatment. Three of these dogs had radiographic evidence of local disease progression at this time but no evidence of pulmonary metastases. One of these dogs was euthanized 3 months later due to spinal metastases; one suffered a pathologic fracture and did not receive her 8^th^ treatment but underwent amputation without adjuvant chemotherapy. This dog survived 491 days and was euthanized following rapid onset of hindlimb weakness and neurological deficits associated with a suspected spinal tumor; no postmortem examination was performed. The third dog (dog 003) showed subsequent stabilization of local disease after initial progression and returned one year later when disease progression was noted on radiographs (Figure 3; Video S1 [baseline] and Video S2 [post]). The dog was euthanized after 864 days due to unexplained lameness on the contralateral limb. Of the three dogs that did not show local disease progression at the time of the eighth Lm-LLO-HER2 administration, one (dog 005) developed pulmonary metastatic disease which waxed and waned over the next 10 months before steadily progressing (Figure 4; Video S3 [baseline] and Video S4 [post]); one developed radiographic signs of local disease progression after receiving 2 booster vaccinations, and underwent amputation and 4 doses of adjuvant carboplatin with no further Lm-LLO-HER2 treatments; he died without clinical evidence of OSA on day 1,660. The other dog (dog 007) showed evidence of local disease progression after receiving 5 booster vaccinations (Figure 5; Video S5 (baseline) and Video S6 (post) and data not shown) which slowly progressed resulting in euthanasia at day 1,013. Lameness scores indicated that most dogs showed no significant progression of their lameness over the initial course of Lm-LLO-HER2 administration unless they suffered a pathologic fracture (Figure S2). Two long-term (LT) survivors showed evidence of pseudoprogression of the primary tumor at the time of V4 and V6 (unscheduled assessment) which showed radiographical improvement approximately 3 months later in each case.

Video S1. Dog 003_Baseline (left proximal humeral lesion)

Video S2. Dog 003_Day 557 (left proximal humeral lesion)

Video S3. Dog 005_Baseline (right distal tibia lesion) Note the visible osteosarcoma lesion on the medial aspect of the right distal tibia.

Video S4. Dog 005_Day 335 (right distal tibia lesion) Note the visible osteosarcoma lesion on the medial aspect of the right distal tibia.

Video S5. Dog 007_Baseline (right distal femur lesion)

Video S6. Dog 007_Day 375 (right distal femur lesion)

**Figure 6:**
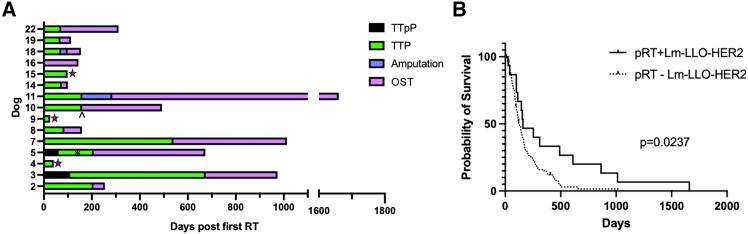
Survival outcomes for dogs with appendicular osteosarcoma (A) Swimmer’s plot for vaccinated dogs with primary appendicular OSA. TTpP, time to pseudoprogression of primary tumor (as determined by radiography); TTP, time to radiographic progression; OST, overall survival time; ∗, time to pseudoprogression of lung lesion; ˆ, time of amputation which coincided with disease progression; red star, euthanized at time of progression. (B) Kaplan-Meier survival curve showing dogs treated with palliative RT (pRT) plus Lm-LLO-HER2 and a historical control group treated with pRT alone. The Mantel-Cox log rank test was used to detect differences in the two survival curves. Tick marks indicate censored patients.

The outcome of each dog is shown in a swimmer plot (Figure 6A). To determine whether addition of Lm-LLO-HER2 to a pRT protocol might provide overall survival benefit, the survival of vaccinated dogs was compared with a previously described historical control group of dogs with appendicular OSA that underwent the same hypofractionated protocol (8 Gy x 2) administered on consecutive days at the Colorado State University Flint Animal Cancer Center.^25^ To serve as an appropriate comparator, dogs in the control group were excluded if they received bisphosphonates or concurrent chemotherapy at the time of pRT; however, those that underwent salvage amputation and/or systemic chemotherapy and/or additional RT after disease progression, were included in the control group because dogs in the Lm-LLO-HER2 dataset were also permitted to receive such salvage therapies. Similar to the Lm-LLO-HER2 treated dogs, dogs in the control group also received concurrent medications as needed for pain management. No dog in either group received losartan or toceranib. The OST of dogs that received Lm-LLO-HER2 plus pRT (*n* = 15, median 159 days) was longer than the OST of dogs in the control group (*n* = 83, median 124 days) (*p* = 0.0237) (Figure 6B). Time to progression was not documented in the control group and as such, a comparison with the vaccinated group could not be made.

Six dogs underwent a complete necropsy, and two dogs underwent a partial necropsy where the affected limb, +/− tumor draining lymph node ± lung lobes were submitted for histopathology. Five of the six dogs that underwent full necropsy had evidence of widespread metastatic disease. The 6^th^ dog was euthanized following a pathologic fracture shortly after his first Lm-LLO-HER2 administration and had no evidence of metastatic disease. One dog that underwent a partial necropsy was found to have focal fibrinosuppurative inflammation surrounding the primary lesion from which *Listeria monocytogenes* was cultured and identified as Lm-LLO-HER2 by MALDI-TOF. This patient received her last treatment (#3) 66 days prior to euthanasia. The outcomes of each dog including time to progression, OST, reason for euthanasia and histopathological necropsy results where available are shown in Table S3.

### Gene expression profiling

**Figure 7:**
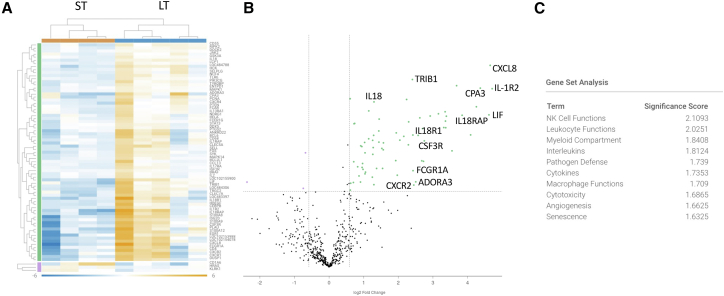
Gene expression analysis of PBMCs at baseline nCounter analysis of PBMCs of long term (LT) survivors versus short term (ST) survivors at baseline, prior to radiation therapy. (A) Heatmap showing differentially expressed genes (fold change ≥1.5 and ≤−1.5; *p* ≤ 0.05) between LT and ST survivors. Mean-subtracted log_2_ normalized counts are shown for DEG between LT and ST groups. (B) Volcano plot showing individual differentially expressed genes between the LT and ST survivors. The log ratio of the fold change is displayed on the *x* axis and the negative log of the adjusted *p* value is on the *y* axis. (C) Gene set analysis with global significance scores reported.

**Figure 8:**
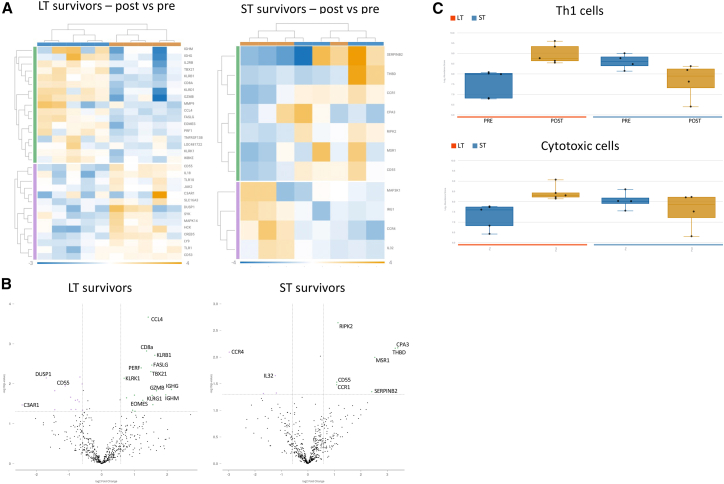
Effects of Lm-LLO-HER2 on gene expression in PBMCs nCounter analysis of PBMCs at the time of the fourth Lm-LLO-HER2 administration compared to baseline in LT and ST survivors. (A) Heatmap showing genes that are differentially expressed genes (fold change ≥1.5 and ≤−1.5; *p* ≤ 0.05) following Lm-LLO-HER2 administration in LT (left) and ST (right) survivors. Blue bar represents pre-treatment values; orange bars represent post-treatment values. Mean-subtracted log_2_ normalized counts are shown for DEG between baseline and fourth vaccination time point. (B) Volcano plots showing individual genes that are differentially expressed following Lm-LLO-HER2 administration for LT and ST survivors. The log ratio of the fold change is displayed on the *x* axis and the negative log of the adjusted *p* value is on the *y* axis. (C) Cell-type profiling within PBMCs for LT (red bar) and ST (blue bar) survivors at baseline and at the fourth vaccination time point.

Gene expression profiles of PBMCs were evaluated at baseline (prior to pRT) and at treatment 4 for 9 dogs and again at treatment 8 for 5 dogs, where RNA of sufficient quality for gene expression profiling (GEP) was available. Dogs were divided into long-term survivors (LT group, *n* = 5 dogs; OST>196 days) and short-term survivors (ST group, *n* = 4 dogs, OST< or = 196 days) based on the median OST of all 15 dogs (196 days). GEP analysis of PBMCs at baseline identified 74 differentially expressed genes (fold change ≥1.5, ≤−1.5-fold change; *p* ≤ 0.05) in LT survivors compared to ST survivors (Figure 7A [heatmap] and B [volcano plot]), with genes involved in NK cell and leukocyte functions, myeloid compartment, interleukins and cytokines, macrophage function, and cytotoxicity being over-expressed in LT survivors (Figure 7C). At the time of the fourth Lm-LLO-HER2 treatment, 31 genes were differentially expressed in LT survivors compared to baseline with 17 genes related to NK cell function, cytotoxicity, and T cell function over-expressed compared to baseline (Figure 8A [heatmap] and B [volcano plot]). In comparison, only 11 genes were differentially expressed after Lm-LLO-treatment in ST survivors, without evidence of up-regulation of cytotoxicity as seen in the LT survivors (Figures 8A and 8B). After deconvolution analysis, a marked increase in signatures associated with Th1 and cytotoxic cells was detected in LT survivors at the time of the fourth Lm-LLO-HER2 administration; however, no increase in these or other leukocyte signatures were observed in ST survivors at this time (Figure 8C). Interestingly, while GEP of PBMCs from LT survivors taken at treatment 8 did not show evidence of NK cell activity or cytotoxicity compared with baseline, gene set analysis revealed significant downregulation of Hedgehog signaling, DNA damage repair and cell cycle/proliferation compared to baseline (Figure S3).

### ELISPOT analysis

To determine whether combination pRT+Lm-LLO-HER2 induces T cell responses against HER2, PBMCs were collected at baseline and every 3 weeks thereafter and evaluated by IFN-γ ELISpot assay using a library of overlapping human HER2 peptides that correspond to the three HER2 domains (EC1, EC2, and IC1) contained within Lm-LLO-HER2. The sequence identity of the human EC1, EC2, and IC1 HER2 domains to canine domains is 89%, 93%, and 98%, respectively. Sufficient baseline PBMCs remained after GEP analysis for evaluation in 6 dogs (5 LT survivors and 1 ST survivor) by IFN-γ ELISpot (Figure S4). Responses (defined as >2-fold increase over background) against EC1, EC2, and/or IC1 HER2 domains were identified in 0/6, 3/6, and 1/6 dogs respectively prior to pRT and Lm-LLO-HER2 treatment. Over the course of 8 treatments, Lm-LLO-HER2 induced 2-fold or greater increases in IFN-γ responses compared to baseline against the EC1, EC2, and IC1 domains of HER2 in 5/6, 5/6, and 5/6 dogs respectively. The one dog that failed to induce detectable HER2-specific IFN-γ responses (dog 005) was an LT survivor that exhibited signs of pseudoprogression of both primary and metastatic lesions throughout the course of the trial (Figure 3 and data not shown).

## Discussion

Tumor burden and an immunosuppressive TME pose significant barriers to the success of immunotherapies in advanced loco-regional solid malignances. However, in this setting, the addition of RT that induces immunogenic cell death can alter the TME to a more favorable immune-permissive state and increases the susceptibility of neoplastic cells to immune-mediated killing, and might be critical for immunotherapeutic success.^33^ Recent bulk RNA sequencing (RNA-seq) data from human and canine patients with primary appendicular OSA have demonstrated that the majority of patients have “cold” tumors, devoid of immune cell infiltrates and these patients have significantly shorter survival times when compared to those with immune-enriched, “hot” tumors.^34^ Low dose RT (<3 Gy) modulates the TME and promotes anti-tumor immunity through multiple mechanisms including the release of damage-associated molecular patterns (DAMPs) and pro-inflammatory cytokines which activate dendritic cells and promote T cell priming, upregulate adhesion molecules on vasculature and lymphatic endothelium increasing immune cell trafficking and the elimination of radiation sensitive cells including regulatory T cells.^35^^,^^36^^,^^37^ In mouse models as well as in a small cohort of human patients with advanced solid tumors low dose RT promoted the infiltration of CD4^+^ Th1 cells into immunologically cold tumors in a process that was shown to be dependent upon IFN-α and IFN-γ.^37^ The optimal radiation dose and schedule that achieves maximum immunological effect is unknown and repeat pRT during Lm-LLO-HER2 treatment or beyond might be valuable to elicit immunity against new epitopes that develop over time. Future mechanistic studies are needed to evaluate the effects of different dosing schedules on the TME of primary OSA lesions.

In this pilot clinical study, we hypothesized that combination pRT with Lm-LLO-HER2 would be safe and lead to enhanced local tumor control. The scientific rationale for this combinatorial sequence was to first induce immunogenic cell death, release TAA and inhibit MDSCs in the TME using ionizing radiation. The potent adjuvant effect of Lm-LLO-HER2 then leads to activation of APCs and production of cytokines that support priming of HER2 specific T cells, boost existing T cell responses against other endogenous TAA released from the irradiated tumor and promote epitope spreading. Our results indicate that combination pRT followed by Lm-LLO-HER2 immunotherapy in the absence of systemic chemotherapy can lead to clinical and radiographic arrest of primary appendicular OSA and may promote abscopal effects that control microscopic metastases and increase overall survival in a subset of patients. Interestingly, LT survivors displayed certain immunological characteristics which distinguished them from ST survivors both before treatment and in response to treatment. Although the number of patients in this study is small, GEP of PBMCs prior to treatment suggested that pre-existing innate, cytotoxic immune activity and inflammation was associated with favorable clinical outcome. Furthermore, LT survivors showed a distinct up-regulation of cytotoxic NK and T cell responses to Lm-LLO-HER2 administration, which was not seen in ST survivors, suggesting that immune fitness and the ability to respond immunologically to Lm-LLO-HER2 is associated with a favorable clinical outcome. Similar findings regarding baseline immune status and immune response to Lm-LLO-HER2 administration were revealed in a large multi-center clinical trial where Lm-LLO-HER2 was administered to dogs with OSA following amputation and chemotherapy to prevent metastatic disease.^15^ Dogs with pre-existing immune activity and with robust immune responses to Lm-LLO-HER2 administration experienced prolonged disease free intervals and OST compared to dogs that did not respond immunologically to Lm-LLO-HER2 administration.^15^ These findings suggest an opportunity exists to stratify patients according to their ability to respond to an immunological stimulus, with those patients deemed to be immunologically fit, streamlined to receive Lm-LLO-HER2. Further investigation is required to determine the most appropriate immune function test that correlates with immune fitness and immunotherapeutic response.

Interestingly, in this study gene set analysis of transcriptomic profiling of PBMCs from LT survivors at the time of the eighth Lm-LLO-HER2 administration did not show sustained cytotoxic responses but instead revealed significant down regulation of genes involved in cell cycle and cell proliferation, which could indicate a waning of anti-tumor immunity in the periphery despite clinical benefit being maintained. Hedgehog signaling was also downregulated in LT survivors. The Hedgehog pathway regulates genes involved in many biological processes including cell proliferation, survival, and cell cycle and it has been shown to play a role in the development and progression of OSA.^38^ Targeted inhibition of the Hedgehog pathway inhibits tumor cell proliferation and metastasis and is being explored as a novel therapeutic strategy for OSA.^39^ In peripheral T cells, Hedgehog signaling reduces activation and proliferation of mature T cells and inhibits T cell cytotoxicity.^40^ Hedgehog signaling was also recently shown to promote M2 polarization of TAMs which suppresses CD8^+^ T cell recruitment to the TME, and to enhance MDSC recruitment to the TME via TGF-β signaling and secretion of CCL2.^41^^,^^42^ As such, it can be hypothesized that down regulation of this pathway might augment T cell mediated anti-tumor immunity. Future studies are needed to determine the mechanism by which Hedgehog signaling is downregulated in LT survivors following immunotherapy and confirm its effects within the TME and metastatic niche.

Combination therapy was found to be safe with only transient, low-grade AEs at the time of Lm-LLO-HER2 administration as previously reported.^14^^,^^15^ Wound dehiscence occurred in 3/15 dogs and is a potential concern in patients undergoing surgical biopsy and RT either concurrently or within one week of each other. The presence of Lm-LLO-HER2 within the primary tumor of one dog in this study is in line with previous reports.^43^^,^^44^ This homing property toward hypoxic tumor tissue and establishment of a bacterial nidus in the immune suppressed TME can be therapeutically exploited to enhance anti-tumor immune responses as well as deliver cytotoxic payloads that can control metastatic disease.^45^^,^^46^ Only 2 of the 15 enrolled dogs had elevated serum ALKP at screening and both dogs had short OSTs, consistent with prior literature indicating elevated ALKP at diagnosis is associated with shorter OST in dogs with appendicular OSA.^47^ Over-expression of HER2 has also been associated with more aggressive disease in some studies of pediatric OSA, while others show no correlation of HER2 expression with patient outcome.^48^^,^^49^ Here, only 10/15 dogs had sufficient tumor tissue available at baseline for HER2 assessment, and there was no statistical correlation between HER2 expression scores, HER2-specific T cell responses as determined by ELISPOT and OST (Table S1). One LT survivor that demonstrated radiographic pseudoprogression of both primary and metastatic lesions following Lm-LLO-HER2 treatment had no evidence of treatment-induced HER2-specific IFN-γ responses. One explanation for this is the adjuvant effect of Lm-LLO, which does not require linkage to a target antigen to boost existing TAA responses and promote epitope spreading.^10^ Further, the effect of Lm-LLO-HER2 on reducing the proportion of Tregs and MDSCs could contribute to HER2 independent anti-tumor activity but this was not evaluated in this study. The majority of patients treated in this study eventually succumbed to their disease and were found to have widespread metastases at multiple different and less common sites including visceral organs (liver, spleen, jejunum, and kidney) as well as the more common pulmonary parenchyma. Future studies to investigate tumor evolution, target antigen mutation, and progressive immune dysfunction leading to loss of immunotherapy-associated tumor control are warranted.

This study has some limitations. The effects of combination therapy on the TME were not evaluated due to the risk of iatrogenic fracture with a second biopsy post-RT. Future studies that evaluate neoadjuvant RT plus Lm-LLO-HER2 prior to amputation and incorporate pre- and post-amputation tumor analyses are needed to better understand the mechanisms by which combination therapy modulates the TME and enhances anti-tumor immunity. Furthermore, exploratory studies to identify correlative biomarkers of clinical response in peripheral blood and within the tumor are also warranted. We used a published protocol for pRT given on 2 consecutive days 3 days prior to the first dose of Lm-LLO-HER2.^24^^,^^25^ The dose, timing and fractionation of ionizing radiation influence its effects on the TME, and it is likely that the optimal RT and combination immunotherapy protocol will vary depending on the immunotherapeutic strategy and the tumor-type targeted. Future prospective studies will be needed to identify the optimal regimen and explore correlative biomarkers of therapeutic immune response. Cellular therapies, checkpoint inhibitors, cytokines, vaccines, oncolytic viruses, and TLR agonists are all being explored in combination with RT underscoring the perceived value of radiation in enhancing responses to immunotherapeutics.

One-quarter of canine OSA patients that have undergone standard of care amputation develop gross pulmonary metastatic disease prior to their third carboplatin administration suggesting intrinsic carboplatin resistance.^50^ Our results in this small pilot study support the further exploration of neoadjuvant combination RT and immunotherapeutic strategies prior to standard of care amputation or in cases where surgical resection is not possible. They also support the evaluation of combination RT + Lm-LLO-HER2 administration in pediatric patients with incomplete tumor resections, or in cases where surgical resection is not possible. Further, they raise an interesting question of whether standard of care, adjuvant, and systemic chemotherapy in the setting of minimal residual disease could be replaced by adjuvant immunotherapeutic strategies to reduce the incidence and progression of metastatic disease in canine and eventually human patients with OSA.

## Materials and methods

### Eligibility criteria and study design

The primary objective of this study was to determine the safety and effectiveness of combination pRT plus Lm-LLO-HER2 to delay primary tumor progression and metastatic disease and prolong OST in dogs with appendicular OSA. The exploratory objective was to determine the effects of combination therapy on systemic anti-tumor immunity.

Treatment naive dogs with a high suspicion of OSA based on signalment, history, clinical signs, physical examination, and radiographic findings, with no evidence of gross metastatic disease, were eligible for screening at the University of Pennsylvania’s School of Veterinary Medicine. The owners of these dogs had elected not to pursue standard of care amputation and chemotherapy for reasons, such as concurrent orthopedic disease, large patient size, and perceived reduction in mobility and quality-of-life issues following amputation and/or cost.

To determine study eligibility, dogs underwent a thorough physical examination, CBC, CS, and urinalysis (UA). Cardiac status and function were determined by echocardiography and serum cardiac troponin I levels. Thoracic radiographs were performed to rule out the presence of pulmonary metastatic disease and 2-view radiographs of the affected limb were obtained. Only systemically healthy dogs with no evidence of cardiac disease or pulmonary metastatic disease, and no evidence of pathologic fracture were eligible to undergo bone biopsy to confirm the diagnosis of OSA. Dogs with a histopathological diagnosis of appendicular OSA and a life expectancy of at least 2 months were eligible for enrollment. HER2 expression within the primary tumor was not an eligibility-criterion and where there was sufficient tumor tissue, HER2 scores were determined retrospectively. A previously described cohort of dogs with a high suspicion of primary appendicular OSA based on age, breed, radiographic findings ± cytology or biopsy that received 2 × 8Gy as palliative RT were used as a comparator group for overall survival.^25^

The study schema is outlined in Figure 1. All eligible dogs were treated with pRT plus Lm-LLO-HER2, there was no placebo group. Eligible dogs received 2 doses of 8 Gy radiation at the site of the primary bone tumor on 2 consecutive days followed 3 days later by systemic administration of the first of 8 planned Lm-LLO-HER2 treatments. Only dogs that received 2 doses of pRT plus at least one dose of Lm-LLO-HER2 were considered evaluable. Lm-LLO-HER2 was administered once every 3 weeks for a total of 8 treatments followed by an optional single booster treatment every 2 months thereafter for dogs that continued to do well clinically. Treatment with chemotherapy or bisphosphonates was not part of the study protocol. Thirty minutes prior to Lm-LLO-HER2 infusion, all dogs received the 5HT3 antagonist ondansetron (0.2 mg/kg i.v.) and the H1 receptor antagonist, diphenhydramine (2 mg/kg i.m.). Lm-LLO-HER2 was kept frozen at −80°C prior to use. At the time of administration, Lm-LLO-HER2 was thawed at room temperature, diluted in 200 mL of 0.9% NaCl and administered intravenously as a constant rate infusion (CRI) over 30 min. All dogs received a fixed dose of 3.3 × 10^9^ CFU of Lm-LLO-HER2. The dose chosen was the highest dose evaluated and shown to be safe when administered i.v. after amputation and chemotherapy, in a phase I clinical trial of dogs with spontaneous OSA.^14^ Temperature, heart rate, heart rhythm, pulse quality, respiratory rate, respiratory effort, and blood pressure were monitored for 6 h post-treatment. In instances where body temperature rose above 103^o^F patients received intravenous crystalloids (Plasmalyte) until their temperature returned to below 103^o^F. Dogs were treated as outpatients. All dogs received a 3-days course of amoxicillin starting 72 h after vaccination to eliminate any remaining *Listeria* bacteria and a 7-days course of S-adenosylmethionine (SAMe) to provide antioxidant support to the liver.

Dogs underwent a full physical exam and were re-staged (CBC, CS, and 3-view thoracic radiographs and 2-view limb radiographs) at day 63 (treatment #4), day 147 (treatment #8), and every two months thereafter until humane euthanasia due to clinical disease progression. Echocardiography was performed at baseline, day 63, day 147, and every two months thereafter at the time of booster treatments. Parameters assessed included left ventricular fractional shortening (LVFS), left ventricular internal dimension in diastole (LVIDd), and left ventricular internal dimension in systole (LVIDs). LVIDd and LVIDs were normalized to body weight as previously described.^51^ Serum cardiac troponin I levels were evaluated at baseline and immediately prior to each Lm-LLO-HER2 administration in the initial series and at each booster administration.

### Lameness assessment

Dogs underwent a full physical exam and were video recorded for lameness assessment at each visit. Dogs were recorded from the side and from the front, walking and trotting at the time of screening, at each Lm-LLO-HER2 administration and every 2 months thereafter to assess clinical response to treatment. Lameness videos were randomized using Research Randomizer, assigned a three-letter code and evaluated by a board-certified veterinary surgeon (KA) who was blinded to the patient ID number and the evaluation time point. A lameness score based on a 1–5 grading scheme was assigned to each patient at each time point where 0 = clinically sound, 1 = barely detectable lameness, 2 = mild lameness, 3 = moderate lameness, 4 = severe lameness, and 5 = non weight bearing lameness. Dogs were allowed to remain on, or initiate analgesics and NSAIDs as needed, throughout the course of the clinical trial, according to standard of care practice.

Where possible, dogs that died during the course of the study underwent necropsy. The presence and location of metastatic disease were recorded and where possible, histopathology and immunohistochemistry were performed to confirm the diagnosis of metastatic OSA and evaluate HER2 status, respectively.

### Toxicity

Toxicities were graded according to the Veterinary Co-operative Oncology Group- Common Terminology Criteria for Adverse Events V2 (VCOG-CTCAE).^52^

### Definitions

Baseline was defined as the date of study enrollment (screening visit). Time to progression was defined as the number of days between the first RT treatment and either sustained clinical progression as determined by an increase in lameness score or sustained radiographic progression of the primary lesion or appearance of pulmonary metastatic lesions that did not regress on subsequent radiographs. The OST was defined as the number of days between the first RT treatment and death. Pseudoprogression is defined as the time point of radiographic progression of the primary tumor that was subsequently shown to improve or resolve on the next radiographic assessment, or the time point at which pulmonary lesions appeared that improved or resolved on the next radiographic assessment, using the time of first RT treatment as day 0. Given that immunotherapy can lead to pseudoprogression of disease, patients continued to receive Lm-LLO-HER2 if their clinical signs were stable or improved and if they were not experiencing significant adverse effects from Lm-LLO-HER2 treatment.

### Ethics statement and regulatory approvals

This study was approved by the University of Pennsylvania’s Institutional Animal Care and Use Committee (Protocol number 804911) and signed owner consent was required prior to enrollment. The use of recombinant DNA was approved by the University of Pennsylvania’s Institutional Biosafety Committee.

### *Lm*-LLO-HER2 manufacture

Lm-LLO-HER2 consists of the *dal*, *dat*, *Act-A* deleted mutant strain of *Lm* (*Lm-ddA*) transfected with the pADV164 plasmid that expresses two 2 extracellular domain fragments (EC1 and EC2) and one intracellular domain fragment (IC1) of human HER2 which together contain the majority of HLA-A2 restricted immunodominant epitopes, fused to a truncated listeriolysin O (LLO).^13^ There is no antibiotic resistance expression cassette and the pADV164 plasmid which contains the bacillus p60 *dal* gene is maintained within the mutant *Lm* via auxotrophic complementation. Vaccines were manufactured by Vibalogics GmbH (Cuxhaven, Germany) and stored at −80°C prior to use.

### Immunohistochemistry

Histopathological assessment of primary and metastatic tumors was performed by a board-certified veterinary pathologist (J.B.E). For HER2 staining, 5 micron sections of formalin-fixed paraffin-embedded tissues underwent antigen retrieval and staining with a rabbit anti-human HER2/neu antibody (Neu(c-18):sc-284, Santa Cruz Biotechnology) or a rabbit IgG isotype (Universal Negative Control serum, NC498, Biocare Medical) as previously described.^14^ Bound antibody was detected using the Universal LSAB 2/HRP kit (Streptavadin-Biotin2 System; Agilent Technologies Inc.). Tissues were stained with 3,3′-diaminobenzidine solution (DAKO) and counterstained with hematoxylin. Bright field images were acquired using a Nikon Digital Sight DS-Fi1 color camera and a NIS-Element BR3.0 for image analysis. Where possible, tissue sections were evaluated and scored for HER2 positivity based on the percentage of HER2^+^ neoplastic cells (<10% = 1, 10%–50% = 2, >50% = 3) and HER2 staining intensity (weak = 1, moderate = 2, strong = 3) within 10 hpf for each tissue section. A combined HER2 score was obtained by multiplying the scores for HER2 positivity by HER2 staining intensity.

### ELISPOT

ELISpot analysis was performed as previously described.^14^ Briefly, cryopreserved PBMC from each indicated time point were thawed, rested overnight at 37°C and then stimulated for 5 days with 2.0 μM pools of overlapping human HER2 peptides (11 mers overlapping by 5 amino acids) that represent the EC1, EC2, and IC1 domains of HER2 present in OST31-164, plus rhIL-2 (Invitrogen) or left un-stimulated in the presence of rhIL-2. Cells were harvested, washed, and counted. IFN-γ ELISpot assays were performed using a canine IFN-γ ELISpot assay kit (R&D Systems). Briefly, 0.1–3 × 10^5^ stimulated cells were incubated with 2.5 μM of EC1, EC2, or IC1 peptide pools or no peptide (to determine background counts) for 24 h. All assays were performed in duplicate and the average number of spots are reported. Plates were developed according to the manufacturer’s instructions. Spots were counted using a CTL-Immunospot analyzer (C.T.L). Samples that had background counts of 0 were assigned a count of “1” to enable fold increase following peptide stimulation to be determined.

### Gene expression profiling of PBMCs

Cryopreserved PBMCs taken prior to RT (baseline), at day 63 (treatment #4), and at day 147 (treatment #8), were thawed, washed twice in complete T cell medium^53^ and twice in 1× PBS and RNA was extracted using the RNeasy Mini or RNeasy Micro Kit (QIAGEN) as per manufacturer’s instructions. RNA quantification was determined using Qubit broad range or high sensitivity assay and RNA quality was determined using the Agilent TapeStation. Samples were hybridized with gene-specific reporter and capture probes (nCounter® Canine IO panel, NanoString Technologies) and processed. Data was acquired on the NanoString nCounter platform. The Canine IO panel comprises 780 immuno-oncology-related target genes, including those involved in immune cell abundance, cytokine and chemokine signaling, checkpoint signaling, interferon signaling, and tumor-specific targets. 20 internal reference control genes are also included. Binding density, detection limits, positive controls, and housekeeping gene counts were measured as part of quality control and data analysis was performed using the Rosalind® platform for nCounter Analysis software. Sample-specific normalization factors were applied to normalize raw data and were calculated using housekeeping genes and the “geNorm” algorithm.^54^ Differential gene expression was calculated using the Rosalind® platform and implementing a generalized linear model on count data. Cell abundance scores for leukocyte subsets were generated using the Cell Type Profiling algorithm on the Rosalind® platform. Dynamic selection was performed to identify signature marker genes stably and specifically expressed in each cell type. Scores for each given cell type were calculated as the arithmetic mean of the log2-transformed normalized expression of all genes associated with that cell type.

### Statistical analysis

The Kaplan-Meier method was used to estimate survival outcomes and the Mantel-Cox log rank test was used to determine differences in OST between the vaccinated group and the historical control group. Statistical significance was set at α = 0.05 and the statistical analysis was performed using commercially available software (GraphPad Prism for Windows, Version 7, San Diego, CA, USA).

## Data and code availability

Data generated in this study are available within the article and its Supplementary Data files. All other data will be made available from the authors upon request.

## Acknowledgments

This study was supported in part by Advaxis who supplied the Lm-LLO-HER2 and the Mason Cancer Research Fund. Gene expression analysis was performed at the Genomics Facility at the Wistar Institute by Sonali Majumdar and Tran Nguyen. Nucleic acid QA/QC was performed by the Center for Host Microbial Interactions at the University of Pennsylvania. We would like to acknowledge Dr. Lilian Duda for her expertise in radiation oncology and treatment of the patients. We thank the Veterinary Clinical Investigations Center at the University of Pennsylvania School of Veterinary Medicine, particularly Angie Cosey, CVT, Heather Scavello, CVT and Samantha Kean, CVT. We would like to thank all of the owners and canine patients that participated in the trial. The content of this publication does not necessarily reflect the views or policies of the Department of Health and Human Services, nor does mention of trade names, commercial products, or organizations imply endorsement by the U.S. Government. The funders had no role in study design, data collection and analysis, decision to publish, or preparation of the manuscript.

## Author contributions

N.J.M., conceptualization, resources, data curation, formal analysis, supervision, investigations, writing – original draft, and writing-review and editing; J.G., resources, investigation, data curation, formal analysis, and writing-review and editing; M.M., resources, investigation, data curation, formal analysis, writing-review and editing; J.R., resources, investigation, data curation, formal analysis, and writing-review and editing; K.A.A., resources, investigation, data curation, formal analysis, and writing-review and editing; F.G., resources, data curation, formal analysis, investigation, visualization, methodology, and writing–review and editing; J.E., resources, data curation, formal analysis, investigation, visualization, methodology, and writing–review and editing; L.O., resources, software, data curation, formal analysis, investigation, visualization, methodology, and writing–review and editing; A.H., resources, data curation, software, formal analysis, methodology, and writing–review and editing; D.H.T., resources, investigation, formal analysis, data curation, methodology, and writing–review and editing; Y.P., conceptualization, resources, methodology, and writing-review and editing.

## Declaration of interests

N.J.M. and Y.P. are inventors on U.S. patents number US20150297702A1 “Compositions and methods for prevention of escape mutation in the treatment of Her2/neu over-expressing tumors” and CA2940646A1 “Compositions and methods for the treatment of HER2 over-expressing tumors”.

Y.P. and N.J.M. are named inventors on patents no. 9,017,660, “Compositions and methods for prevention of escape mutation in the treatment of HER2/neu over-expressing tumors,” and no. 10,016,617, “Combination immunotherapy and radiotherapy for the treatment of HER2-positive cancers.” N.J.M. has equity in OS Therapies and serves as an advisor for OS Therapies and OS Animal Health Corp.

## Supplemental information

Document S1. Tables S1–S3 and Figures S1–S4

Document S2. Article plus supplemental information
