# Supplementary material for: Combined radiation and Listeria immunotherapy induces cytotoxic immunity that correlates with improved outcome in dogs with osteosarcoma
Source: Mol Ther Oncol. 2026 May 21;34(2):201243. doi: 10.1016/j.omton.2026.201243 (PMC13264224; doi:10.1016/j.omton.2026.201243)
Supplement: Document S1. Tables S1–S3 and Figures S1–S4 [file mmc1.pdf]

OMTON, Volume *34*

Supplemental information

Combined radiation and *Listeria* immunotherapy induces cytotoxic immunity that correlates with improved outcome in dogs with osteosarcoma

Nicola J. Mason, Josephine Gnanandarajah, Martha MaloneyHuss, Jennifer Reetz, Kimberly A. Agnello, Falon Gray, Julie Engiles, Lauren Olenick, Andrew Hart, Douglas H. Thamm, and Yvonne Paterson

SUPPLEMENTAL TABLES

Table S1. Patient signalment and tumor characteristics.

Dog number	Age (years)	Breed	Sex	Primary Tumor Location	Subtype	ALKP status	HER2/Neu intensity	HER2/Neu distribution	HER2/Neu combination score	No. of doses administered (n=booster doses)
002	9	Italian Spinone	MC	Proximal humerus	Osteoblastic	normal	N/A	N/A	N/A	8
003	6	Great Pyrenees	FS	Proximal humerus	Osteoblastic	normal	N/A	N/A	N/A	8 (+1)
004	9	Irish Setter	FS	Distal radius	Osteoblastic	elevated	3	2	6	2
005	8	Golden Retriever	MC	Distal tibia	Osteoblastic	normal	N/A	N/A	N/A	8 (+4)
007	7	German Shepherd	MC	Distal femur	Osteoblastic	normal	3	1	3	8 (+10)
008	7	Great Dane	MC	Distal radius	Osteo/Tele	normal	3	2	6	5
009	7	Greyhound	MC	Proximal humerus	Fibroblastic	normal	3	2	6	1
010	9	Mixbreed	FS	Proximal humerus	Osteoblastic	normal	2	1	2	7
011	9	Mixbreed	MC	Distal tibia	Osteoblastic	normal	3	1	3	8 (+2)
014	7	Great Pyrenees	FS	Distal radius	Osteoblastic	normal	1	2	2	3
015	7	Greyhound	FS	Proximal humerus	Osteoblastic	normal	N/A	N/A	N/A	6
016	9	Saluki	MI	Distal radius	Fibroblastic	normal	N/A	N/A	N/A	5
018	8	Rottweiler	FS	Proximal humerus	Osteoblastic	elevated	3	2	6	6
019	10	Rottweiler	FS	Distal femur	Chondroblastic	normal	3	3	9	3
022	5	Irish Wolfhound	FS	Distal radius	Osteoblastic	normal	3	3	9	4

MC, male castrated; FS, Female spayed; M, male intact; N/A, not available

Table S2. Adverse events as defined by VCOG-CTCAE v2

Abnormality	Grade	Parameters	Number of dogs (total)
**Constitutional:**			
Pyrexia	1	103.5-104	3
	2	>104-105.5	10
Vomiting	1	<24hr	9
Nausea	1	no intervention required	8
**Cardiovascular:**			
Arrhythmias	1	Asymptomatic	1
	2	Non-urgent intervention	1
Tachycardia	1	HR > 140 BPM	5
	2	HR>180 BPM	0
	3	HR>200 BPM	1
Hypotension	1	Systolic < 100 mmHg	1
Hypertension	1	Systolic > 160 mmHg	14
**Hematological:**			
Thrombocytopenia	1	100,000/ul to <LLN	3
	2	50,000 to 99,000/ul	1
**Biochemical:**			
ALKP	1	>ULN to 2.5x ULN	1
	2	>2.5 to 5x ULN	1
	3	>5 to 20 x ULN	1
ALT	1	>ULN to 1.5x ULN	1
AST	1	>ULN to 1.5x ULN	3
Cardiac troponin 1		>0.2 ug/L	0
**Post biopsy complications**			
Wound dehiscence	1	Asymptomatic	0
	2	Symptomatic, medical intervention	2
	3	Symptomatic, invasive intervention	1

Table S3. Outcomes and necropsy results

Dog no	Reason for euthanasia	Post mortem	Primary site	Metastatic site	Histopathology	TTP	OS
002	Spinal pain	Y	Proximal humerus	Lungs, liver, spleen, omentum, kidney, rib, vertebrae (T5, L1 & L5) and subcutis	Primary: OSA with multifocal myofiber necrosis, atrophy and loss; metastatic lesions: all metastatic foci contain variable amounts of tumor osteoid, with exception of some microscopic pulmonary metastatic foci, and one from the spleen and right kidney. Cellular morphology consistent with OSA	207	254
003	Progressive lameness in unaffected limb	N	Proximal humerus	Unknown	N/P	675	864
004	Primary tumor progression	Y	Distal Radius	Right pre-scapular LN, right axillary LN, lungs, Heart, spleen	Primary: OSA with abundant necrosis and hemorrhage, regional extensive dermal edema, ulceration and serosuppurative crust; LN- focal metastatic OSA, marked drainage reaction with lymphofollicular hyperplasia and medullary sinus erythrocytosis and hemosiderin-laden macrophages; spleen multifocal complex nodular hyperplasia and metastatic OSA, lungs-multifocal metastatic OSA, marked regional neutrophilic interstitial pneumonia with extensive necrosis and hemorrhage	41	41
005	Primary tumor progression	Y	Distal Tibia	Lungs, jejunum	Lungs: multifocal intravascular and parenchymal OSA-pulmonary emboli – with mural invasion and hemorrhage, alveolar hemorrhage, edema and histiocytosis; jejunum – metastatic mural OSA	208	611
007	Primary tumor progression	Y	Distal Femur	Visceral pleura (left caudal and accessory lobes), kidneys, lung	Primary: modest inflammatory infiltrate with mature lymphocytes, plasma cells and fewer histiocytes expand the periosteum of distal left femur and adjacent neoplasm; kidneys – small number of inflammatory infiltrate surround metastatic nodules; lung-pedunculated nodules of primarily acellular neoplastic woven bone with few dispersed aggregates of neoplastic cells and no significant inflammation	540	1013
008	Primary tumor progression + pathologic fracture	Y	Distal Radius	Lung	Lungs: multifocal perivascular lymphohistiocytic infiltrate surrounding metastatic nodules, edema, hemorrhage and congestion	85	159
009	Primary tumor progression + pathologic fracture	Y	Proximal humerus	None	Primary: Severe subacute to chronic regionally extensive epiphyseal fracture of the articular surface	26	26
010	Pathologic fracture + amputation	N	Proximal humerus	Unknown	N/P	159	491
011	Geriatric decline	N	Distal Tibia	Unknown	N/P	160	1660
014	Primary tumor progression + pathologic fracture	N	Distal Radius	Unknown	N/P	75	100
015	Suspect pathologic fracture	N	Proximal humerus	Unknown	N/P	99	99
016	Hemoabdomen – liver and splenic mass	N	Distal radius	Unknown	N/P	144	144
018	Hemothorax	N	Proximal humerus	Unknown	N/P	72	154
019	Lethargy and inappetence	Partial	Distal Femur	Lung (right cranial, caudal and middle lobes)	Primary – chondroblastic OSA – mitotic index 31, large areas of necrosis, necrotic neutrophils and foci of hemorrhage, no significant inflammatory infiltrate; lungs – multiple individual to coalescing densely cellular, partially encapsulated neoplastic nodules, MI=10 in some areas, low to moderate numbers of lymphocytes and few plasma cells surround small blood vessels in the intervening parenchyma, small dispersed aggregates of lymphocytic inflammation infiltrate deeper within larger neoplastic nodules. Minimal inflammation is associated with nodules less then 1mm, large tumor emboli multifocally occlude pulmonary arteries	69	113
022	Primary tumor progression and weakness	Partial	Distal Radius	Lung (multiple nodules and 8-10cm mass in L caudal lung lobe; omentum and liver nodules, rib mass	Histopath available for Primary tumor and draining LN: The neoplasm is composed of sheets bundles and streams of ovoid to spindle shaped cells the cells have a fairly extensive amount of eosinophilic cytoplasm and round, oval eccentrically located nuclei with prominent nucleoli. There is moderate anisocytosis and anisocytosis and mitosis average 1-3 per high power field. The neoplastic cells often surround an amorphous eosinophilic matrix which in some areas has mineralized. Draining (axillary) lymph node: Drainage reaction with marked lymphoid hyperplasia	72	311

SUPPLEMENTAL FIGURES

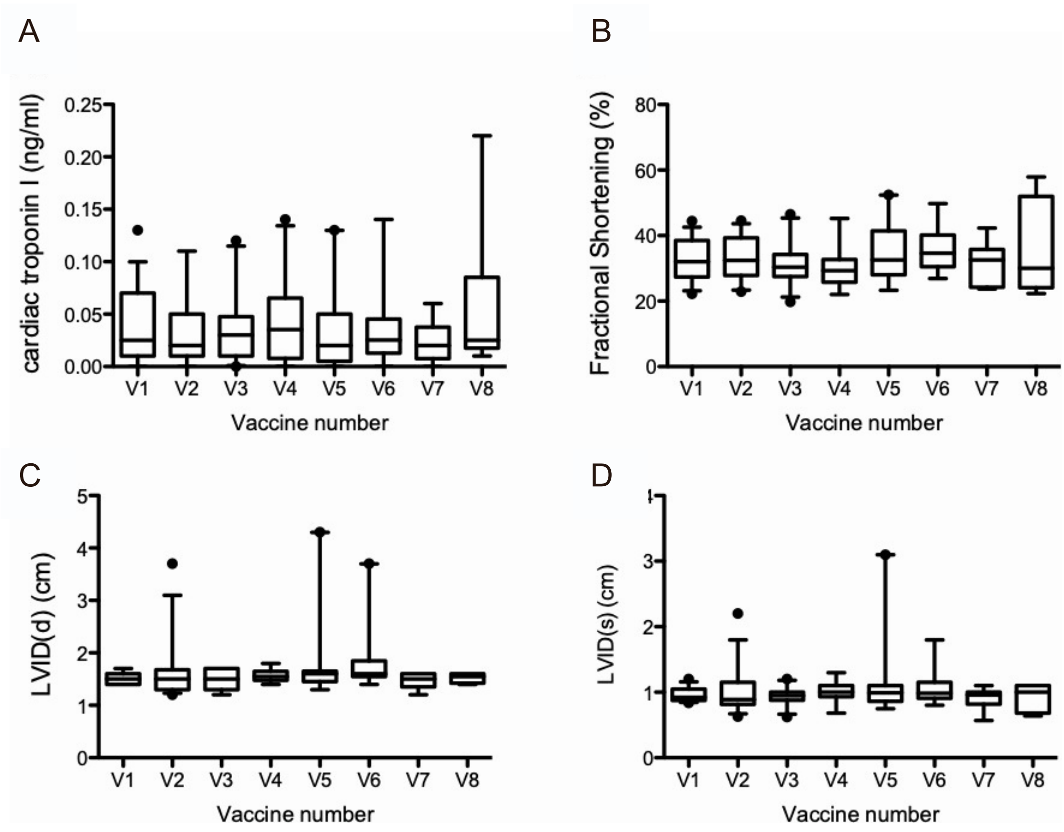

Figure S1. Lm-LLO-HER2 does not adversely affect cardiac function. LVID(d) left ventricular internal dimension in diastole; LVID(s) left ventricular internal dimension in systole.

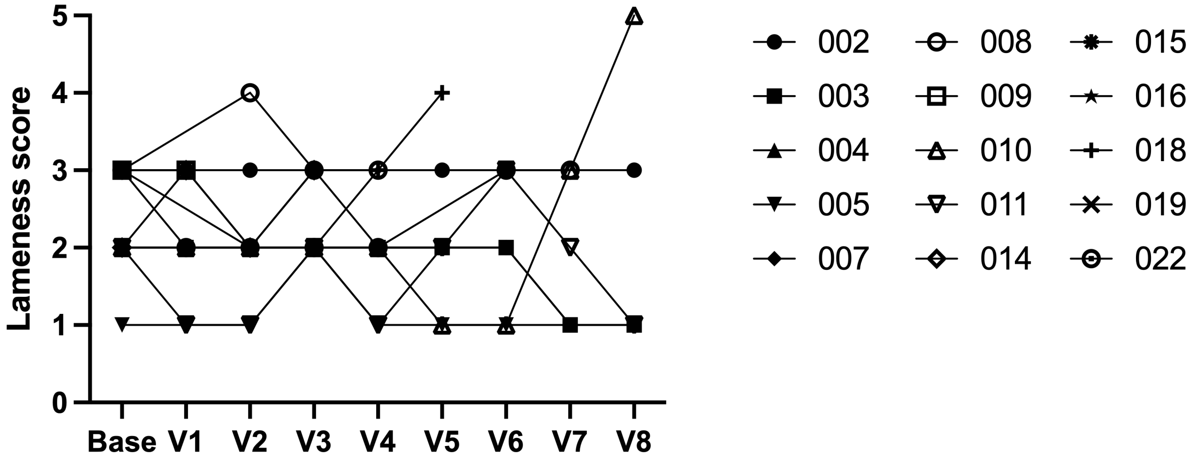

Figure S2. Lameness scores assigned to study patients at timepoints indicated. Lameness scores for each dog at each time point are plotted. Base refers to lameness score assessment prior to radiation therapy.

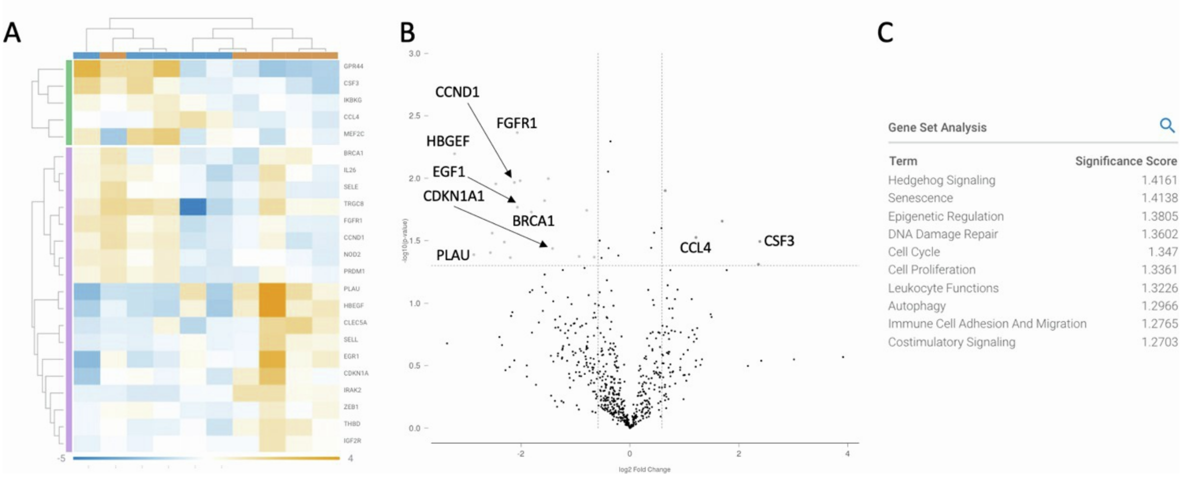

Figure S3. Gene expression profiling of PBMCs at baseline and at V8. A. Gene expression analysis of PBMCs taken at the time of the eighth *Lm*-LLO-HER2 administration (blue) compared to baseline (prior to pRT; orange) are shown for LT survivors. **B.** Volcano plots of differentially expressed genes at the eighth *Lm*-LLO-HER2 administration compared to baseline for LT survivors. **C.** Gene set analysis of differentially expressed genes.

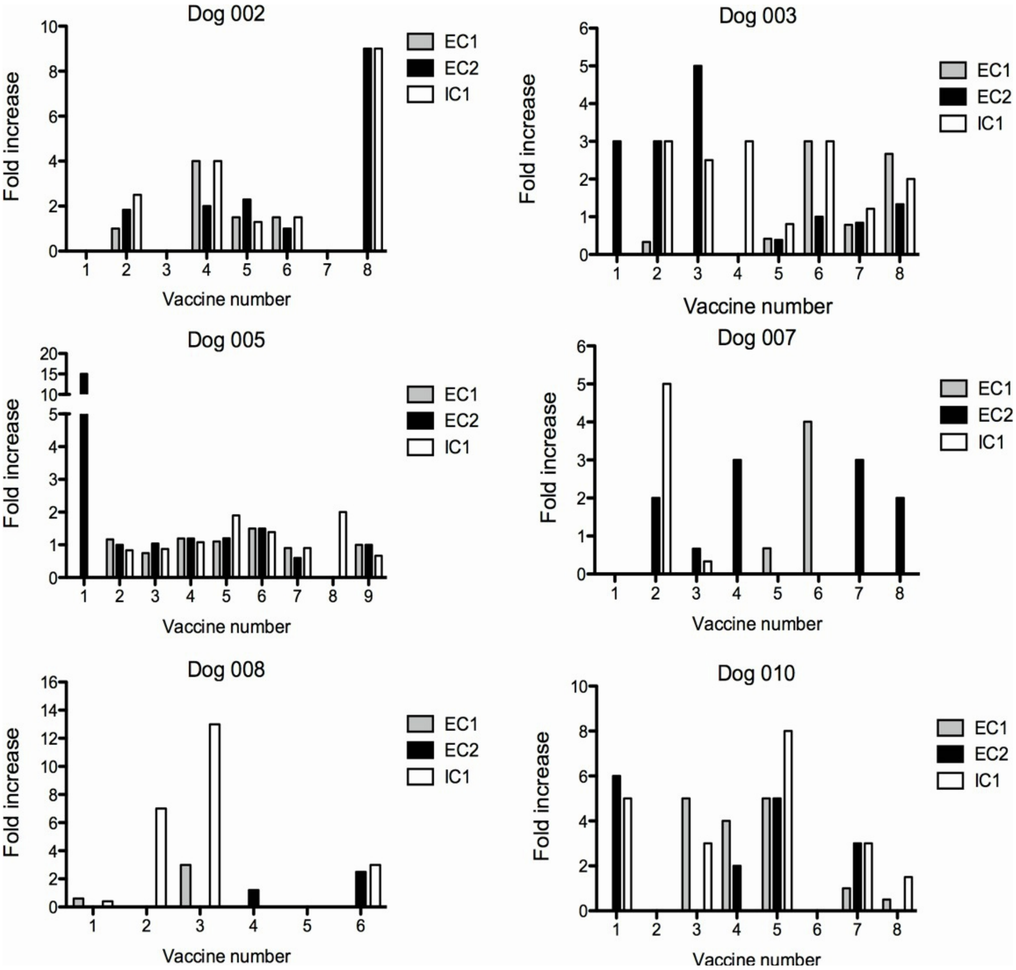

Figure S4. HER2-specific IFN-γ responses from 6 dogs treated with pRT and *Lm*-LLO- HER2. PBMCs were collected at baseline (prior to radiation and *Lm*-LLO-HER treatment) (vaccine 1 timepoint) and at every *Lm*-LLO-HER2 thereafter. PBMCs were incubated with a library of human overlapping HER2 peptides that encompass the extracellular domain 1 (EC1), extracellular domain 2 (EC2) or intracellular domain 1 (IC1) of HER2 and were then analyzed for IFN-γ production by ELISpot assay. Fold increase in spot number over background is reported for each overlapping peptide library.

SUPPLEMENTAL VIDEO FILES

Video S1_Dog 003_Baseline (left proximal humeral lesion)

Video S2_Dog 003_Day 557 (left proximal humeral lesion)

Video S3_Dog 005_Baseline (right distal tibia lesion). Note the visible osteosarcoma lesion on the medial aspect of the right distal tibia

Video S4_Dog 005_Day 335 (right distal tibia lesion). Note the visible osteosarcoma lesion on the medial aspect of the right distal tibia

Video S5_Dog 007_Baseline (right distal femur lesion)

Video S6_Dog 007_Day 375 (right distal femur lesion)
